# Relationship of alien species continues in a foreign land: The case of *Phytophthora* and Australian *Banksia* (Proteaceae) in South African Fynbos

**DOI:** 10.1002/ece3.9100

**Published:** 2022-07-14

**Authors:** Axola Qongqo, Felix Nchu, Sjirk Geerts

**Affiliations:** ^1^ Centre for Invasion Biology Department of Conservation and Marine Sciences Cape Peninsula University of Technology Cape Town South Africa; ^2^ South African National Biodiversity Institute Kirstenbosch Research Centre Cape Town South Africa; ^3^ Department of Horticultural Sciences Cape Peninsula University of Technology Bellville South Africa

**Keywords:** *Banksia ericifolia*, floriculture, invasion, invasive alien plants, minimum inhibitory concentration, phytopathogens, *Phytophthora cinnamomi*

## Abstract

Fungal invasions only recently started to receive more attention in invasion biology. This is largely attributed to little or non‐existent information about these inconspicuous organisms. Most invasion hypotheses focus on factors that increase invasion success; few try to explain why invasions fail. Here we hypothesize that a host–pathogen relationships can limit the invasiveness of an alien plant species in a novel range. To test this, we investigate whether the invasiveness of the Australian genus of Proteaceae, *Banksia,* in South Africa is determined by the alien and major invasive phytopathogen, *Phytophthora cinnamomi*. The presence of *P. cinnamomi* in *Banksia* root and soil was evaluated using morphological and molecular techniques. Isolates were cultured onto selective media and polymerize chain reactions and internal transcribing spacers were used for identification. Acetone leaf extracts of 11 *Banksia* spp. were screened for antimicrobial activity against *P. cinnamomi*, using the minimum inhibitory concentration assay. A total of 3840 *Banksia* individuals from seven localities were surveyed. *Phytophthora  cinnamomi* was consistently isolated from *Banksia* species root and soil samples. Out of the 12 *Banksia* species that were screened for antimicrobial activity, four introduced species, *B. burdettii*, *B. coccinea*, *Banksia hookeriana*, and *B. prionotes* and the invasive *B. integrifolia* and *B. ericifolia* exhibited relatively high antimicrobial activity against *P. cinnamomi* (strain 696/12). We show that the phytopathogen in the native range has similar impact in the novel range and in doing so may limit invasion success of *Banksia* species with low antimicrobial activity.

## Graphical abstract

Most invasion hypotheses focus on factors that increase invasion success, few try to explain why invasions fail. Here we hypothesize that a host‐pathogen relationships can limit the invasiveness of an alien plant species in a novel range. To test this, we investigate whether the invasiveness of the Australian genus of Proteaceae, *Banksia*, in South Africa is determined by the alien phytopathogen, *Phytophthora cinnamomi*. A total of 3840 *Banksia* individuals from seven localities were surveyed. *Phytophthora cinnamomi* was consistently isolated from *Banksia* species roots and soil samples. Out of the twelve *Banksia* species that were screened for antimicrobial activity, four introduced and two invasive species exhibited high antimicrobial activity against *P. cinnamomi*. We show that the phytopathogen in the novel range has similar impacts than in the native range and in doing so limits invasion by *Banksia* species with low antimicrobial activity. We provide an additional tool with which *Banksia* species that pose a high invasion risk ‐ whether already invasive or just introduced ‐ due to lower susceptibility to *P. cinnamomi,* can be prioritised in legislation and for management.

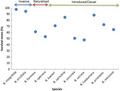

## 1 INTRODUCTION

Biological invasions have received substantial attention over the past few decades (Downey & Richardson, 2016; Kitching et al., 2011). This is important since invasive species contribute to native species loss and extinction (Sax et al., 2002), erode natural capital, compromise the delivery of ecosystem goods and services and threaten economic productivity (Richardson & van Wilgen, 2004). But the fundamental question of why some introduced species become more abundant and widespread than others still remains only partially answered (Keane & Crawley, 2002; Kolar & Lodge, 2001; van Kleunen et al., 2010). There are various factors that influence the degree of invasion in an ecosystem; these include—among others—fires, historical habitat modification, propagule pressure, release from herbivores, human usage, pollinators, and habitat suitability (Geerts & Adedoja, 2021; Geerts et al., 2016; Geerts, Botha, et al., 2013; Honig et al., 1992; Mangachena & Geerts, 2019; Sundaram et al., 2015). Many hypotheses have been put forward to explain these phenomena (reviewed in Catford et al., 2009). Interestingly, the testing of these hypotheses mostly focuses on factors that increase invasion success (Catford et al., 2009). However, understanding the factors that could slow down or inhibit an invasion by an alien plant species in a novel range has received considerably less attention. One such hypothesis—known as the Enemy Inversion Hypothesis—is that the natural enemies of alien species are also introduced into the novel range but are less effective, allowing for invasion. In contrast, if the enemy is effective, that should limit or even prevent an invasion.

Here we propose a hypothesis largely similar to the Enemy Inversion Hypothesis, which we term the Global Enemy Hypothesis, which states that if a species is attacked by an alien enemy in its native range, it will similarly be attacked in a novel range where this enemy is also present and alien. Here, we test this hypothesis by considering the invasiveness of the Australian genus of *Banksia* (Proteaceae) in South Africa and whether it is impeded by the alien phytopathogen, *Phytophthora cinnamomi*. *P. cinnamomi* is an oomycete phytopathogen that causes diseases to several plant species and has a massive impact in certain natural ecosystems, forestry, and agriculture globally (Cahil et al., 2008). *P. cinnamomi* causes symptoms such as wilting, canker, and dieback on host plants (Tommerup et al., 1999).

Invasion by alien plants is an expanding problem in South Africa with about 10 million ha (8.28%) of South Africa's landscapes are affected by invasive alien plants (van Wilgen et al., 2020). Australian plant species are prominent invaders in South Africa (van Wilgen et al., 2020). While a number of genera (such as *Leptospermum*, *Acacia*, *Hakea*) have been in the country for a long time, more recent introductions from genera such as *Anigozanthus* (Erckie et al., 2022; Le Roux et al., 2010), *Melaleuca* and *Banksia* have followed (Geerts, Moodley, et al., 2013; Jacobs et al., 2015; Matthys et al., 2022; Moodley et al., 2016).

The genus *Banksia* belongs to the family Proteaceae and has the highest number of introduced species from this plant family in South Africa (Moodley et al., 2014). *Banksia* species were initially introduced to South Africa for floriculture in the 1970s and about 15 species are present of which some have become invasive (Moodley et al., 2014). The genus *Banksia* presents an ideal study group to understand the importance of phytopathogens in limiting plant invasions, firstly, we have a good understanding of *Banksia* invasions in South Africa (Geerts, Moodley, et al., 2013; Moodley et al., 2013, 2014, 2016; Richardson et al., 1990). Secondly, within this genus there are non‐invasive, naturalized and invasive species in South Africa (invasion defined as per Richardson et al., 2011). Lastly, susceptibility to phytopathogens differs between the different *Banksia* species.

Preliminary surveys of *Banksia* spp. in South Africa revealed that some species are potentially parasitized by multiple pathogens and that there might be variability in the susceptibility between *Banksia* species to these pathogens. Plants are immobile and cannot physically escape their natural enemies; therefore, they synthesize a wide range of phenolic compounds as defense mechanisms against pathogen attack (Bell, 1980). These phenolic compounds can act as antimicrobial agents against phytopathogens or bacteria (Lattanzio et al., 2006). Reports from Australia have linked the phytopathogen, *P. cinnamomi* to the dieback of some *Banksia* species (Davis et al., 2014; Shearer & Dillon, 1996). Similarly, the most damaging plant pathogen of native South Africa Proteaceae is *P. cinnamomi* (Von Broembsen, 1984, 1985; Wood, 2017). In the late 1980s, Richardson et al. (1990) conducted a post‐border risk assessment and predicted that four *Banksia* species (*B. burdettii*, *B. coccinea*, *B. hookeriana*, *B. prionotes*) are high risk species and are likely to become invasive in the Cape Floristic Region (CFR). Prior to this study, Von Broembsen (1984) found these four species to be parasitized by the phytopathogen *P. cinnamomi* in the Southwestern Cape of South Africa. The potential risk of invasiveness can be guided by invasive species risk analyses (Kumschick et al., 2020; Pheloung et al., 1999), but whether invasiveness by *Banksia* species is potentially hampered by closely associated phytopathogens, remains to be tested.

In this study, we address some of these issues and aim to understand the importance of oomycetes in limiting plant invasions by using the genus *Banksia* in the CFR as a case study. For this we, (1) determine *Banksia* mortality in South Africa, (2) determine whether *Banksia* mortality is caused by *P. cinnamomi*, (3) assess whether *Banksia* resistance to *P. cinnamomi* species relates to invasiveness, and (4) conduct weed risk analyses.

## 2 METHODS

### 2.1 Study area and study species

The study was conducted at seven *Banksia* species localities in the CFR of South Africa (−34° 37′ 48.144“; 19° 41’ 25.0188”) (Table 1). The CFR is categorized by a sub‐Mediterranean climate with cold winters and warm, dry summers. The region is approximately 90,000 km^2^ and contains over 9000 plant species (Collins & Rebelo, 1987;Cowling & Richardson, 1997; Moran & Hoffmann, 2012). The vast majority of plant species in the region are fire prone and thrive on nutrient poor soils (Cowling & Richardson, 1997; Moran & Hoffmann, 2012).

**TABLE 1:** *Banksia* species and *Phytophthora cinnamomi* survey data in the Cape Floristic Region included in this study

Site	Latitude	Longitude	Species	Soil	Roots	Dead	Alive	Survival	pH (KCl)	Soil type
Blomkloof	S34,520694	E 19.794278	*Banksia baxteri*	Yes	Yes	40	64	62%	4.1	LmSa
Blomkloof	S34,520694	E 19.794278	*Banksia speciosa*	Yes	Yes	89	11	11%	5.1	LmSa
Blomkloof	S34,527639	E 19.810472	*Banksia spinulosa*	Yes	Yes	1	99	99%	4.6	LmSa
Blomkloof	S34,527639	E 19.810722	*Banksia formosa*	Yes	Yes	28	72	72%	4.5	LmSa
Blomkloof	S34,523444	E 19.820861	*Banksia serrata*	Yes	Yes	73	27	27%	4.8	Sa
Blomkloof	S34,533335	E 19.773333	*Banksia integrifolia*	Yes	Yes	0	100	100%	5.2	LmSa
Blomkloof	S34,518861	E 19.796167	*Banksia coccinea*	Yes	Yes	9	91	91%	4.8	LmSa
Blomkloof	S34,519028	E 19.796194	*Banksia hookeriana*	Yes	Yes	21	79	79%	4.7	LmSa
Blomkloof	S34,532972	E 19.773306	*Banksia ericifolia*	Yes	Yes	3	97	97%	4.8	LmSa
Blomkloof	S34,518694	E 19.796650	*Banksia prionotes*	Yes	Yes	24	76	76%	4.0	LmSa
Blomkloof	S34,519222	E 19.796444	*Banksia menziesii*	Yes	Yes	0	100	100%	4.6	LmSa
Blomkloof	S34,530947	E 19.732125	*Banksia integrifolia*	Yes	Yes	0	60	100%	5.3	Sa
Viljoens Hof	S34,532661	E 20.030428	*B. ericifolia*	Yes	Yes	2	25	93%	4.5	LmSa
Eenvoud	S34,476794	E 19.7400420	*B. coccinea*	Yes	Yes	26.00	4	13,33%	4.9	Sa
Akkersdrif	S34,353677	E18.819535	*B. integrifolia*	No	No	0	6	100%	3.8	Sa
Eenvoud	S34,479055	E19.738841	*B. baxteri*	Yes	Yes	16.00	24.00	67%	4.5	Sa
Eenvoud	S34,480273	E19.739462	*B. hookeriana*	Yes	Yes	2	92	98%	5	Sa
Eenvoud	S34,480329	E19.739627	*B. prionotes*	Yes	Yes	0	8	100%	4.1	Sa
Eenvoud	S34,476654	E19.739962	*B. serrata*	Yes	No	23	51	69%	4.7	Sa
Eenvoud	S34,475383	E19.741710	*B. formosa*	Yes	Yes	42	68	62%	4.7	Sa
McGregor	S33,998312	E19.762645	*B. speciosa*	Yes	Yes	51	49	49%	4.5	LmSa
McGregor	S33,994841	E19.758957	*B. formosa*	Yes	Yes	22	22	50%	4.7	LmSa
McGregor	S34,001124	E19.764166	*B. coccinea*	Yes	Yes	55	50	48%	4.3	LmSa
Napier	S34,630045	E 19.690283	*B. baxteri*			100	75	43%		
Napier	S34,630012	E 19.690245	*B. menziesii*			39	17	30%		
Napier	S34,630067	E 19.690023	*B. coccinea*			15	127	89%		
Napier	S34,630699	E 19.692081	*B. prionotes*			100	75	43%		
Napier	S34,630716	E 19.691784	*B. prionotes*			96	224	70%		
Napier	S34,520305	E 19.794963	*B. coccinea*			107	147	58%		
Napier	S34,520736	E 19.794137	*B. speciosa*			0	417	100%		
Napier	S34,520375	E 19.794999	*B. coccinea*			28	73	72%		
Napier	S34,527744	E 19.806551	*B. spinulosa*			56	139	71%		
Napier	S34,520375	E 19.794999	*B. coccinea*			0	203	100%		

*Note*: Table is arranged per site, as multiple species were sampled per site. Abbreviations used for soil are: LmSa for Loam Sandy soil, Sa for Sandy soil. Soil and roots columns refer to whether these were sampled for a specific species at a given locality.

*Banksia* localities were obtained from Geerts, Moodley, et al. (2013), Moodley et al. (2014), Moodley et al. (2016), iNaturalist, South African Plant Invaders Atlas and consultations with local experts, farmers, and conservationists. Populations included managed plantations and naturalized populations. During surveys we added localities—which had not been previously recorded—for *B. coccinea*, *B. baxteri*, *B. hookeriana*, *B. prionotes*, *B. serrata*, *B. formosa*, and *B. intergrifolia*.

### 2.2 *Banksia* mortality surveys and soil nutrients

Out of the 14 *Banksia* species occurring in the CFR, the survival percentage of 11 species was determined. Dead banksias were still identifiable. The other three species were either cleared or only occur as a few isolated individuals. To determine plant mortality a minimum of two haphazardly placed quadrats of 50 × 50 m were established per locality for each species.

For soil nutrient analysis, 1 kg of soil was collected—at the stem base of *Banksia* individuals showing necrosis—for 11 *Banksia* species. Samples were sent to a commercial laboratory (Bemlab Pty Ltd, in Somerset West, South Africa) for a complete soil analysis.

### 2.3 *Phytophthora cinnamomi* collection, isolation, identification, and zoospore preparation

For 11 *Banksia* species at seven localities (at some localities there were more than one species) in the CFR (Table 1), 1500 g soil was sampled from the top 10 cm at the bases of *Banksia* individuals showing necrosis. Isolation from soil and diseased plant samples was performed within 48 h of collection. Bait solution was prepared by mixing soil samples (20 g) with 100 ml of sterile deionized water (dH_2_O); the bait solution was mixed for 3 min using a vortex mixer. Sterile *Citrus* sp. leaf sections (0.5 m^2^) were submerged in the bait solution for 3 days. After 3 days, *Citrus* sp. leaf sections were plated onto selective NARP (natamycin. ampicillin, rifamyicin, pentachloronitronzene) agar (Jeffers & Martin, 1986) and incubated in the dark for 5 days at 25°C. Root samples of 1 cm^2^ were dissected, rinsed for 5 min, surface sterilized with 70% ethanol, and left to air dry. Sterile root samples were plated on NARP agar and incubated in the dark for 5 days at 25°C. To obtain pure cultures, 1 cm diameter of solid agar containing actively growing oomycetes was transferred onto clean Petri dish with NARP agar.

Actively growing 1‐cm‐diameter mycelium on NARP agar was transferred to 10% V8 broths. Mycelium of isolates grown in V8 broth was harvested and rinsed with dH_2_O, excess water was removed with a filter paper, and the mycelium was placed in 2‐ml microfuge tubes and lyophilised with VirTris® Advantage BenchTop Tray Lyophilizer (SP Scientific, UK) overnight. The dried mycelium was then transferred into sterile microfuge tubes with two 3‐mm metal beads. This was followed by extraction of the total genomic deoxyribose nucleic acid (DNA) and amplification of target genes. DNA was extracted from the mycelium by adding 60 μl Prepman® Ultra DNA extraction buffer (Applied Biosystems, UK), then they were heated at 96°C and crushed. The Internal Transcribed Spacer regions of the rDNA (ITS1 and ITS2) were amplified using the primers ITS6 (Cooke & Duncan, 1997) and ITS4 (White et al., 1990). Forward and reverse sanger sequences were uploaded and aligned in Geneious v. R6 and consensus sequencing were compared with an internal data set ITS curated for published Oomycetes using Blast.

*Phytophthora cinnamomi* isolate (696/12 12 g) was selected for the minimum inhibitory concentration (MIC) bioassay, and this is attributed to its consistent growth and spread when it was cultured on NARP agar. A diameter (1 cm) of *P. cinnamomi* was cut from the margins of the NARP medium using a sterile cork borer and transferred into a selective medium; 1000 ml Nutrient Broth Merck (Pty.Ltd, South Africa) containing antibiotics (25 mg/ml pimaricin, 100 mg/ml ampicillin, 5 mg/ml rifamycin, 100 mg/ml pentachloronitrobenzene) (Jeffers & Martin, 1986), to allow for zoospore counts, and then incubated for 60 min at 23°C. A hemocytometer was used to count zoospores. The final spore concentration for the MIC was maintained at 1 × 10^5^ cells/ml (Nchu et al., 2010).

Fresh leaf materials were collected from the 11 *Banksia* spp. The leaves were used instead of the roots since harvesting leaves was less destructive to the cultivated commercial plantations, and they were easy to dry and grind to fine powder for extraction and bioassay. Leave material was oven‐dried at 30°C for 5 days. The dried leaves were ground into fine powder using a Jankel and Kunkel Model A10 mill. Ground leaf material (5 g) was extracted with 100 ml of acetone in a glass beaker using a vortex mixer for 15 min and then filtered through Whatman No.1 filter paper. The plant extracts were left to air dry in a fume cabinet overnight at room temperature (22 ± 2°C).

### 2.4 Minimum inhibitory concentration bioassay

The MIC assay, previously described by Eloff (1998) and Nchu et al. (2010), was adopted with modifications. The bioassay was conducted on 12 *Banksia* species with six replicates for each species. The bioassay was conducted using 96‐well microplates; 100 μl of dH_2_0 was added to each well, followed by a serial successive dilution of acetone plant extract (positive control consisted only out of acetone) to obtain an initial concentration of 6 mg/ml for all wells. A concentration of 100 μl of *P. cinnamomi* (10^5^ cells/ml) was added to each well, and finally 40 μl of (0.2 mg/ml of p‐iodonitrotetrazolium (Sigma) dissolved in dH_2_0, was also added to each well. The bio‐reagent p‐iodonitrotetrazolium salt acted as an electron acceptor and displayed a red color due to biological active organisms (Eloff, 1998). Microplates were sealed with plastic and incubated at 25°C in the dark. Minimum inhibitory concentration (MIC) values were recorded periodically every 6 h for 24 h by visually observing a red color indicating biological active organisms. The same protocol was used for the negative control by substituting acetone plant extract with acetone and for the positive control, substituting 480 μg/ml amphotericin b dissolved in acetone. Antimicrobial activity was rated in these four categories: (i) (1 ≤ 3 mg/ml) high antimicrobial activity; (ii) (3.1 ≤ 4 mg/ml) intermediate antimicrobial activity; (iii) (3.6 ≤ 5.9 mg/ml) low antimicrobial activity; and (iv) (≥6 mg/ml) no activity. We present the 18‐h antimicrobial activity data because it best represents the bioassay activity; at 24 h of the bioassay only a few species still showed activity.

### 2.5 Weed risk assessments

We used the Australian Weed Risk Assessment (A‐WRA), developed by Pheloung et al. (1999)—and applied the guidelines of Gordon et al. (2010) for application of this system outside Australia—to evaluate the potential risk posed by *Banksia* species in the CFR. The A‐WRA is an assessment of a species based on its biology, biogeography, history, and ecology. The A‐WRA is a useful system to quickly predict potentially invasive plants. The assessment consists of 49 questions, and each question is awarded points of between −3 and 5. The final answer of the assessment results is based on the possible three outcomes regarding the species; if a species has score <1, it can be introduced, if a species has a score >6, the species cannot be introduced; and lastly if a species outcome score is 1–6 the species requires further evaluation. If the data available in the literature was insufficient to answer all 49 questions a minimum of 10 questions was answered (Moodley et al., 2017).

### 2.6 Statistical analysis

A one‐way ANOVA with a post‐hoc Tukey HSD test was used to determine differences in antimicrobial activity between *Banksia* species. Statistical analysis were done in R statistics ver.3.4.3 (R Core Team, 2015).

## 3 RESULTS

### 3.1 *Banksia* mortality and soil nutrients

**FIGURE 1:**
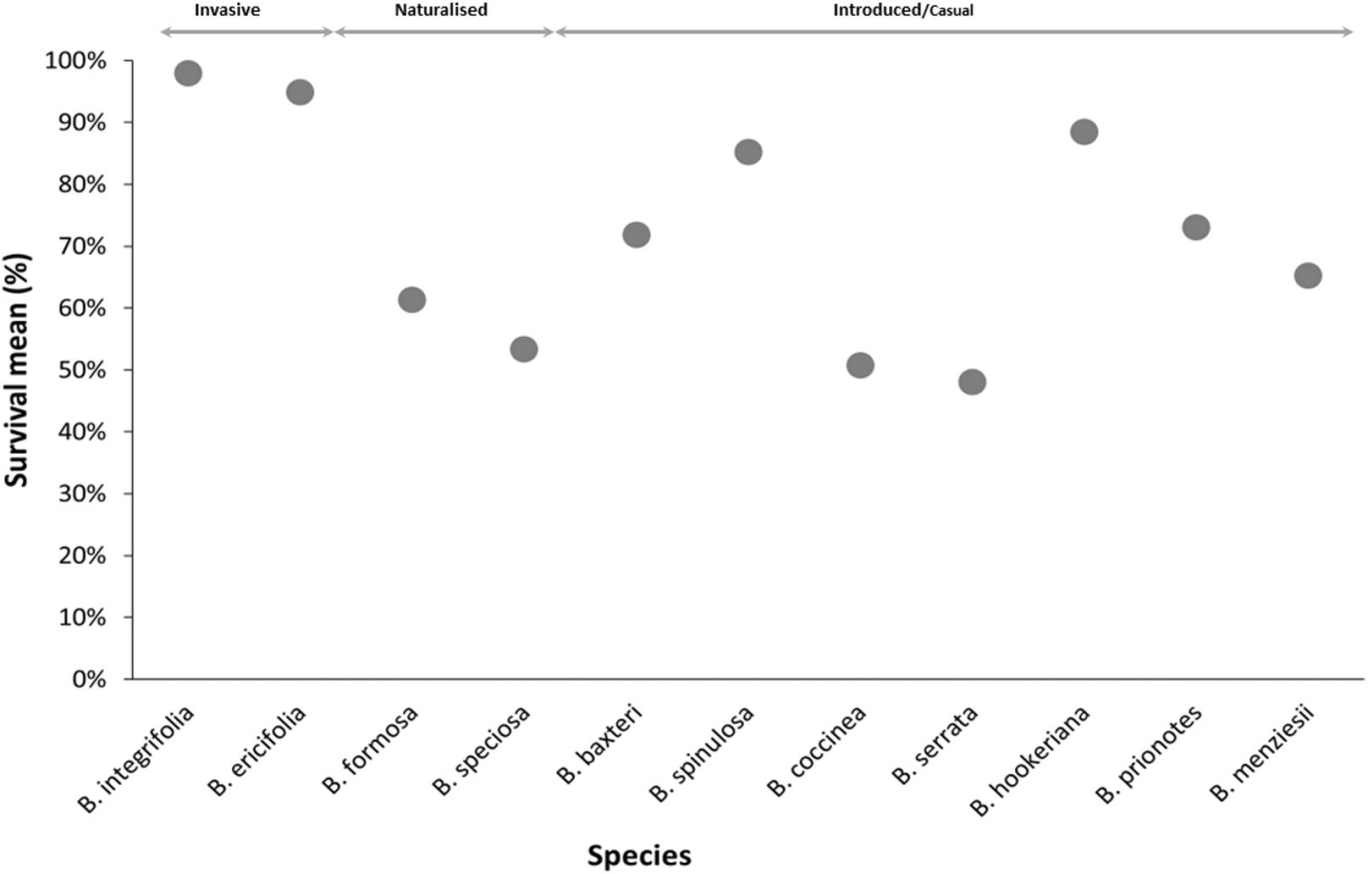
Survival mean (%) of the 11 *Banksia* species at *Phytophthora cinnamomi* infested localities in the Cape Floristic Region. Circles depict means. Species invasion status indicated at the top of the graph

**FIGURE 2:**
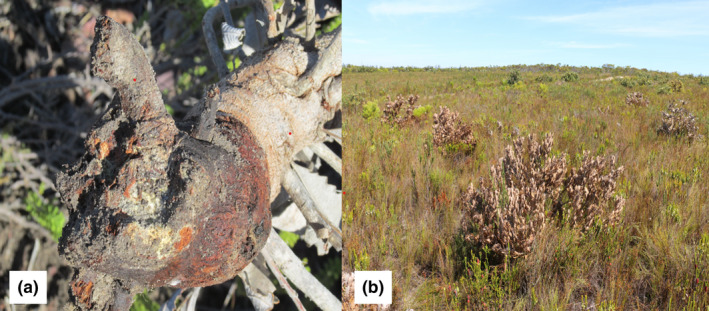
(a) *Phytophthora cinnamomi* infested *Banksia* spp. base, rotten showing “canker”; (b) *P. cinnamomi* infected *Banksia formosa* plants dying

A total of 3840 *Banksia* individuals were surveyed in this study; 1068 individuals were recorded as dead, and 2772 individuals were alive. The survival rate varied between species with the two invasive species, *Banksia integrifolia* (98%) and *B. ericifolia* (95%), having the highest survival rate (Figure 1; Table 1). The survival rate of introduced species varied from high in *B. spinulosa* (85%) and *B. hookeriana* (89%) to *B. serrata* (48%) as the lowest. Dying *Banksia* individuals showed clear symptoms of necrosis and were rotten at the base of the stem (Figure 2a). The two naturalized species, *B. speciosa* (53%) and *B. formosa* (61%), also had relatively high mortality (Figure 2b).

No significant difference in soil nutrients were found between sites, neither was it correlated to *Banksia* mortality (*p* > .05 for all correlations) (Table S1); nonetheless, these baseline data are useful for long‐term monitoring of soil physicochemical properties.

### 3.2 Minimum inhibitory concentration bioassay

**FIGURE 3:**
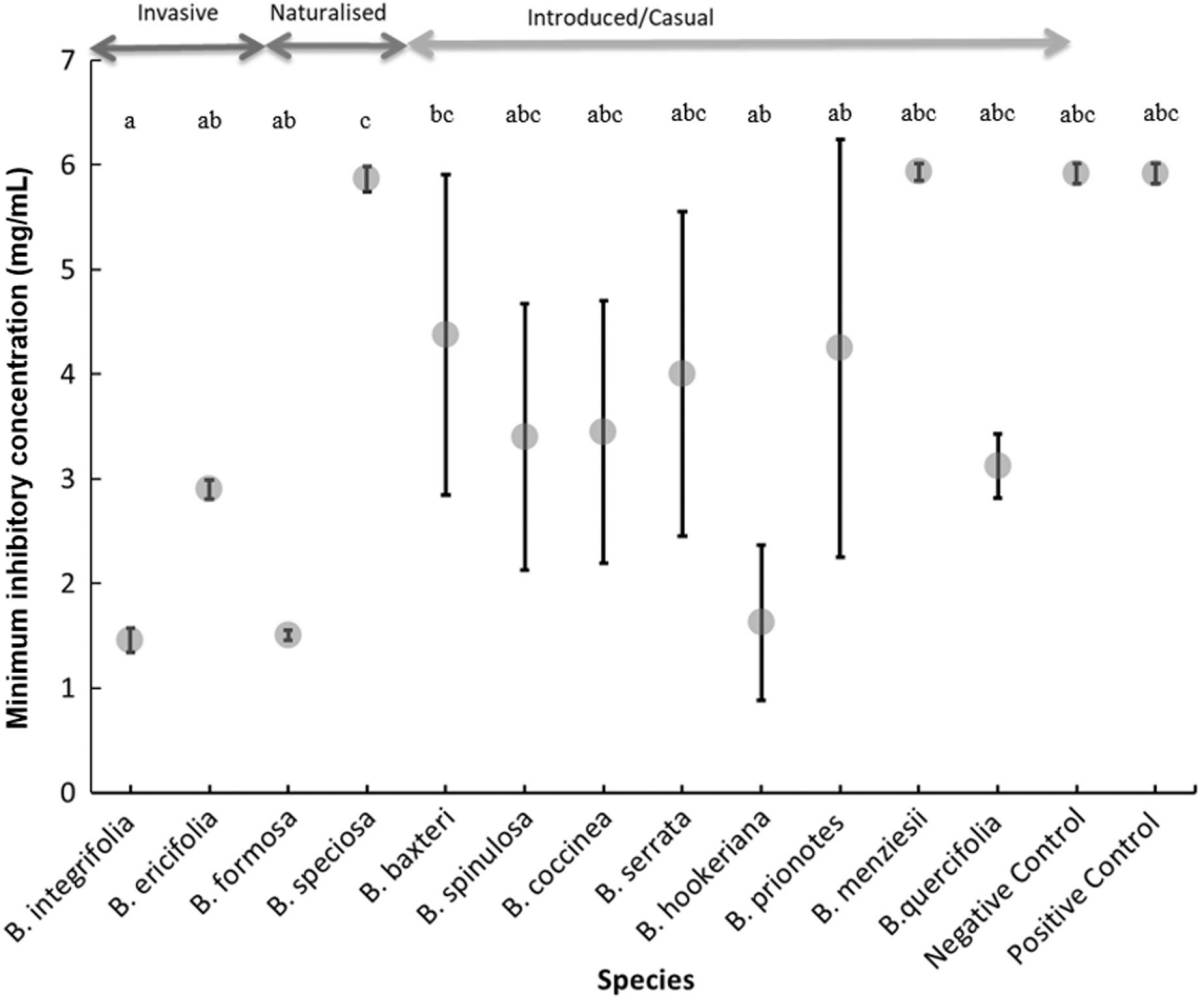
Antimicrobial activity (minimum inhibitory concentration) of *Banksia* species acetone leaf extracts against *Phytophthora cinnamomi* (positive and negative control had no antimicrobial activity after 18 h against *P. cinnamomi*). Dots and lines on the graph depicts maximum and minimum antimicrobial activity. High activity: 0 ≤ 3 mg/ml. Medium activity: 3 ≤ 5 mg/ml. No activity: 5 ≤ 6 mg/ml

The MIC activity was significantly different among *Banksia* species (*F* = 18.2, *df* = 13, *p* < .001). Remarkably, the two invasive species, *B. integrifolia* and *B. ericifolia* together with two introduced species, *B. formosa* and *B. hookeriana* exhibited high antimicrobial activity (growth inhibition), based on the low MIC values, against *P. cinnamomi* (1 < 3 mg/ml) (Figure 3; Table S1). Five species, the naturalized *B. coccinea* and introduced *B. baxteri*, *B. quercifolia*, *B. prionotes*, and *B. spinulosa* showed intermediate antimicrobial activity after 18 h. *Banksia speciosa*, *B. hookeriana*, and *B. menziesii* exhibited little antimicrobial activity. The positive and negative control showed no antimicrobial activity against *P. cinnamomi* after 18 h.

### 3.3 Weed risk assessments

The risk assessment outcome of six species is a reject (score of 6<) (Table A1 in Appendix 1; Table S2). Species with scores of 6 or higher are considered to have a high risk of becoming invasive and cannot be introduced. Two of these species—*B. ericifolia* and *B. integrifolia*—are already listed as invasive, whilst *B. speciosa*, *B. spinulosa*, *B. quercifolia*, and *B. sphaerocarpa* are considered high risk. Eight *Banksia* species require further evaluation (score of 1–6) and can thus not be assigned to potentially invasive or casual; this is largely attributed to insufficient data to assess the risk posed by these species (Appendix 1).

## 4 DISCUSSION

Here we show that large‐scale *Banksia* mortality—across populations and species—in South Africa is caused by *P. cinnamomi*. We isolated *P. cinnamomi* from 90% of the *Banksia* localities we surveyed, which is in conjunction with studies on native species finding *P. cinnamomi* to be common in the CFR (Linde et al., 1999; Von Broembsen, 1984, 1985). *Banksia* species with low or no mortality in the field show high antimicrobial activity in the bioassays, this includes the two invasive species, *B. ericifolia* and *B. integrifolia* (Geerts, Moodley, et al., 2013; Moodley et al., 2013, 2014). In contrast, species with high mortality in the field, such as the naturalized *B. speciosa*, showed little antimicrobial activity. Although Moodley et al. (2013) showed that this species has all the traits to become invasive, these results potentially explain why *B. speciosa* is not a more widespread invader. Consequently, species such as *B. hookeriana* and *B. formosa*, that show high antimicrobial activity but are not invasive now, could do so in future. However, species with low antimicrobial activity should still be treated with caution. The weed risk assessments can provide a guide for this. In fact, according to the weed risk assessments, five species, namely *B. speciosa*, *B. ericifolia*, *B. integrifolia*, *B. quercifolia*, and *B. spinulosa*, pose a high risk of invasion. *B. ericifolia* and *B. integrifolia* are known to be invasive species and *B. speciosa* is an emerging invader (Adedoja et al., 2021). Interestingly, species from Eastern Australia generally are more resistant than those from western Australia (McCredie et al., 1985), but this does not hold across all species in this study (see for example *B. serrata* from eastern Australian).

*Banksia* antimicrobial activity and mortality in the field in the invaded range in South Africa are very similar to the native range of Banksia species (Cahil et al., 2008; Hardham & Blackman, 2018; McCredie et al., 1985; Tommerup et al., 1999; Tynan et al., 1998). Thus, antimicrobial activity is an important factor which may partly explain the difference in species mortality rate and thus invasions success. Interestingly, for one species, *B. hookeriana*, the antimicrobial activity is low in Australia, but we found high antimicrobial activity against *P. cinnamomi* and in the field we observed high survival rate in *P. cinnamomi* infested populations. The reason for *B. hookeriana* being different might be that there is variation within species with large native ranges, or species that are widely used in floriculture and horticulture. Taken together, here we show that the proposed global enemy hypothesis holds and that this largely explains the differences in invasion status of *Banksia* species in South Africa. It is worth noting that soil nutrient levels neither varied significantly among the sampled sites nor correlate with mortality. This strengthens the hypothesis, since mortality is not influenced by differences in soil nutrients.

Indeed, those *Banksia* able to resist *P. cinnamomi* infection tend to become invasive. However, this merely acts as one filter, which together with other filters such as propagule pressure, pollinators and fire, will determine whether a species will invade (Adedoja et al., 2021; Geerts, 2011; Geerts & Adedoja, 2021; Geerts et al., 2017; Le Roux et al., 2020). Fire was observed by Geerts, Moodley, et al. (2013) as an important factor which facilitated invasion success of *B. ericifolia* after a lag phase in the CFR whilst pollinators are not important in predicting invasiveness Moodley et al. (2016). Here, we show that selected *Banksia* species are resistant to *P. cinnamomi* and poses a high risk of invasion in the CFR. Consequently, as a first step to reducing the risk posed by these species, this study suggests that selected *Banksia* species resistant to *P. cinnamomi* (*B. ericifolia*, *B. integrifolia*, *B. hookeriana*, and *B. formosa*) should be prioritized for management and legislation. There are no *Banksia* species listed in South Africa's National Environmental Management: Biodiversity Act (10/2004) AIS regulations (2020 lists). Nevertheless, given the accumulative evidence gathered from previous studies, (Geerts, Moodley, et al., 2013; Moodley et al., 2013, 2014), as well as this study, we recommend that *B. ericifolia* and *B. integrifolia* be listed under NEM: BA; as category 1a or category 2 (permits required) species. *B. spinulosa*, *B. quercifolia*, *B. hookeriana*, and *B. formosa* have shown that they have antimicrobial activity against *P. cinnamomi* and possess invasive traits, and therefore we suggest plantations (and surrounding natural areas) of these species are closely monitored.

In conclusion, several *Banksia* species are of economic importance to the horticultural and floricultural industries. Therefore, we advocate that new introduction are carefully screened with pre‐border risk analyses and that potentially invasive species—as identified by this study—be rejected and other species closely monitored. Species that are highly susceptible to *P. cinnamomi* and pose a low risk of invasion based on the WRA could potentially be safely introduced into the country and still be profitable to grow. The plant pathogens can be treated in plantations and the flowers used for floriculture. Lastly, conducting species MIC bioassay can be used as a reliable tool to predict possible resistance of a plant species to a pathogen. For future research, we recommend in situ and ex situ *P. cinnamomi* inoculation trials on *Banksia* species under different watering regimes.

## AUTHOR CONTRIBUTIONS

**Axola Qongqo:** Conceptualization (equal); data curation (lead); formal analysis (lead); funding acquisition (supporting); methodology (equal); project administration (equal); writing – original draft (lead); writing – review and editing (equal). **Felix Nchu:** Conceptualization (equal); data curation (supporting); funding acquisition (supporting); investigation (equal); methodology (equal); project administration (equal); resources (supporting); software (supporting); supervision (supporting); writing – original draft (supporting); writing – review and editing (equal). **Sjirk Geerts:** Conceptualization (lead); funding acquisition (lead); investigation (supporting); methodology (equal); project administration (supporting); resources (lead); software (equal); supervision (lead); writing – original draft (supporting); writing – review and editing (equal).

## CONFLICT OF INTEREST

The authors declare no conflict of interest.

### OPEN RESEARCH BADGES

This article has earned an Open Data badge for making publicly available the digitally‐shareable data necessary to reproduce the reported results. The data is available at https://datadryad.org/stash/share/meS2dv1vOlSfgUmYnG0mn_fEcUFYikWdfEXvuYZz7MM.

## Supporting information


Appendix S1

Table S1

Table S2

## Data Availability

Data openly available in a public repository that issues datasets with DOI: Dryad https://doi.org/10.5061/dryad.0cfxpnw3j.

## APPENDIX 1

**TABLE A1:** *Banksia* species survival mean, weed risk assessment outcome, and antimicrobial activity of *Banksia* species acetone leaf extracts

Species	Survival mean (%)	A‐WRA score	Outcome	Antimicrobial activity (mg/ml)
*Banksia integrifolia*	98.00	13	Reject	1.45
*Banksia ericifolia*	95.00	11	Reject	2.90
*Banksia coccinea*	51.00	2	Further evaluation	3.45
*Banksia speciosa*	53.00	8	Reject	5.86
*Banksia baxteri*	72.00	1	Further evaluation	4.37
*Banksia spinulosa*	85.00	9	Reject	3.40
*Banksia formosa*	61.00	3	Further evaluation	1.50
*Banksia serrata*	48.00	2	Further evaluation	4.00
*Banksia hookeriana*	88.00	4	Further evaluation	1.62
*Banksia prionotes*	73.00	1	Further evaluation	4.25
*Banksia menziesii*	65.00	5	Further evaluation	5.93
*Banksia quercifolia*	–	7	Reject	3.45
*Banksia burdetti*	–	2	Further evaluation	–^a^
*Banksia sphaerocarpa*	–	8	Reject	–^a^

^a^ No antimicrobial activity analyses were conducted on these species because populations have been removed or could not be found.
