# Supplementary material for: Relationship of alien species continues in a foreign land: The case of Phytophthora and Australian Banksia (Proteaceae) in South African Fynbos
Source: Ecol Evol. 2022 Jul 14;12(7):10.1002/ece3.9100. doi: 10.1002/ece3.9100 (PMC9280440; doi:10.1002/ece3.9100)
Supplement: Supplementary file 1 — Appendix S1 [file ECE3-12--s001.pdf]

Weed Risk Assessment report 20-Oct-17

	**Botanical name:** *Banksia baxteri*			**Outcome:**	0
	**Common name:**			**Score:**	
	**Name of Assessor:**			**Agricultural**	0
	**A. History/Biogeography**			**Environmental**	0
				**Total**	1
A	**1** *Domestication/*	1,01	Is the species highly domesticated? If answer is 'no' go to 2.01		no
C	*cultivation*	1,02	Is species naturalised where grown?		no
C		1,03	Does the species have weedy races?		
	**2** *Climate and*	2,01	Species suited to Australian climates (0-low; 1-intermediate; 2-high)		2
	Distribution	2,02	Quality of climate match data (0-low; 1-intermediate; 2-high)		2
C		2,03	Broad climate suitability (environmental versatility)		
C		2,04	Native or naturalised in regions with extended dry periods		
		2,05	Does the species have a history of repeated introductions outside its natural range?		yes
C	**3** *Weed*	3,01	Naturalised beyond native range		
E	*Elsewhere*	3,02	Garden/amenity/disturbance weed		no
A		3,03	Weed of agriculture/horticulture/forestry		no
E		3,04	Environmental weed		no
		3,05	Congeneric weed		no
	**B. Biology/Ecology**				
A	**4** *Undesirable*	4,01	Produces spines, thorns or burrs		no
C	*traits*	4,02	Allelopathic		no
C		4,03	Parasitic		no
A		4,04	Unpalatable to grazing animals		no
C		4,05	Toxic to animals		
C		4,06	Host for recognised pests and pathogens		no
C		4,07	Causes allergies or is otherwise toxic to humans		no
E		4,08	Creates a fire hazard in natural ecosystems		yes
E		4,09	Is a shade tolerant plant at some stage of its life cycle		
E		4,10	Grows on infertile soils		yes
E		4,11	Climbing or smothering growth habit		no
E		4,12	Forms dense thickets		no
E	**5** *Plant*	5,01	Aquatic		no
C	*type*	5,02	Grass		no
E		5,03	Nitrogen fixing woody plant		no
C		5,04	Geophyte		no
C	**6** *Reproduction*	6,01	Evidence of substantial reproductive failure in native habitat		
C		6,02	Produces viable seed		yes
C		6,03	Hybridises naturally		no
C		6,04	Self-fertilisation		
C		6,05	Requires specialist pollinators		no
C		6,06	Reproduction by vegetative propagation		no
C		6,07	Minimum generative time (years)		
A	**7** *Dispersal*	7,01	Propagules likely to be dispersed unintentionally		no
C	*mechanisms*	7,02	Propagules dispersed intentionally by people		yes
A		7,03	Propagules likely to disperse as a produce contaminant		no
C		7,04	Propagules adapted to wind dispersal		yes
E		7,05	Propagules buoyant		
E		7,06	Propagules bird dispersed		no
C		7,07	Propagules dispersed by other animals (externally)		
C		7,08	Propagules dispersed by other animals (internally)		
C	**8** *Persistence*	8,01	Prolific seed production		yes
A	*attributes*	8,02	Evidence that a persistent propagule bank is formed (>1 yr)		yes
A		8,03	Well controlled by herbicides		
A		8,04	Tolerates or benefits from mutilation, cultivation or fire		yes
E		8,05	Effective natural enemies present in Australia		yes
	**Statistical summary**			Biogeography	0
	**of scoring**		Score partition:	Undesirable attributes	1
				Biology/ecology	0
				Biogeography	5
			Questions answered:	Undesirable attributes	10
				Biology/ecology	17

A = agricultural, E = environmental, C = combined

Weed Risk Assessment report

20-Oct-17

**Botanical name:** *Banksia burdetti*	**Outcome:** 0
**Common name:**	**Score:**
**Name of Assessor:**	**Agricultural** 0
	**Environmental** 0
	**Total** 2

	**A.**	**History/Biogeography**			
A	**1**	*Domestication/ cultivation*	1,01	Is the species highly domesticated? If answer is 'no' go to 2.01	no
C			1,02	Is species naturalised where grown?	no
C			1,03	Does the species have weedy races?	
	**2**	*Climate and Distribution*	2,01	Species suited to Australian climates (0-low; 1-intermediate; 2-high)	2
			2,02	Quality of climate match data (0-low; 1-intermediate; 2-high)	2
C			2,03	Broad climate suitability (environmental versatility)	no
C			2,04	Native or naturalised in regions with extended dry periods	
			2,05	Does the species have a history of repeated introductions outside its natural range?	no
C	**3**	*Weed Elsewhere*	3,01	Naturalised beyond native range	
E			3,02	Garden/amenity/disturbance weed	no
A			3,03	Weed of agriculture/horticulture/forestry	no
E			3,04	Environmental weed	
			3,05	Congeneric weed	
	**B.**	**Biology/Ecology**			
A	**4**	*Undesirable traits*	4,01	Produces spines, thorns or burrs	no
C			4,02	Allelopathic	no
C			4,03	Parasitic	no
A			4,04	Unpalatable to grazing animals	
C			4,05	Toxic to animals	
C			4,06	Host for recognised pests and pathogens	no
C			4,07	Causes allergies or is otherwise toxic to humans	
E			4,08	Creates a fire hazard in natural ecosystems	yes
E			4,09	Is a shade tolerant plant at some stage of its life cycle	
E			4,10	Grows on infertile soils	yes
E			4,11	Climbing or smothering growth habit	no
E			4,12	Forms dense thickets	no
E	**5**	*Plant type*	5,01	Aquatic	no
C			5,02	Grass	no
E			5,03	Nitrogen fixing woody plant	no
C			5,04	Geophyte	no
C	**6**	*Reproduction*	6,01	Evidence of substantial reproductive failure in native habitat	
C			6,02	Produces viable seed	yes
C			6,03	Hybridises naturally	
C			6,04	Self-fertilisation	yes
C			6,05	Requires specialist pollinators	
C			6,06	Reproduction by vegetative propagation	no
C			6,07	Minimum generative time (years)	
A	**7**	*Dispersal mechanisms*	7,01	Propagules likely to be dispersed unintentionally	no
C			7,02	Propagules dispersed intentionally by people	yes
A			7,03	Propagules likely to disperse as a produce contaminant	no
C			7,04	Propagules adapted to wind dispersal	yes
E			7,05	Propagules buoyant	
E			7,06	Propagules bird dispersed	no
C			7,07	Propagules dispersed by other animals (externally)	
C			7,08	Propagules dispersed by other animals (internally)	
C	**8**	*Persistence attributes*	8,01	Prolific seed production	
A			8,02	Evidence that a persistent propagule bank is formed (>1 yr)	
A			8,03	Well controlled by herbicides	
A			8,04	Tolerates or benefits from mutilation, cultivation or fire	
E			8,05	Effective natural enemies present in Australia	

**Statistical summary of scoring**	Score partition:	Biogeography	0
		Undesirable attributes	2
		Biology/ecology	0
	Questions answered:	Biogeography	4
		Undesirable attributes	8
		Biology/ecology	12

A = agricultural, E = environmental, C = combined

Weed Risk Assessment report

20-Oct-17

	**Botanical name:** *Banksia coccinea*			**Outcome:**	0
	**Common name:**			**Score:**	
	**Name of Assessor:**			**Agricultural**	0
				**Environmental**	0
	**A. History/Biogeography**			**Total**	0
A	**1** *Domestication/*	1,01	Is the species highly domesticated? If answer is 'no' go to 2.01		no
C	*cultivation*	1,02	Is species naturalised where grown?		no
C		1,03	Does the species have weedy races?		
	**2** *Climate and*	2,01	Species suited to Australian climates (0-low; 1-intermediate; 2-high)		2
	Distribution	2,02	Quality of climate match data (0-low; 1-intermediate; 2-high)		2
C		2,03	Broad climate suitability (environmental versatility)		yes
C		2,04	Native or naturalised in regions with extended dry periods		
		2,05	Does the species have a history of repeated introductions outside its natural range?		yes
C	**3** *Weed*	3,01	Naturalised beyond native range		no
E	*Elsewhere*	3,02	Garden/amenity/disturbance weed		no
A		3,03	Weed of agriculture/horticulture/forestry		no
E		3,04	Environmental weed		no
		3,05	Congeneric weed		no
	**B. Biology/Ecology**				no
A	**4** *Undesirable*	4,01	Produces spines, thorns or burrs		no
C	*traits*	4,02	Allelopathic		no
C		4,03	Parasitic		no
A		4,04	Unpalatable to grazing animals		
C		4,05	Toxic to animals		no
C		4,06	Host for recognised pests and pathogens		no
C		4,07	Causes allergies or is otherwise toxic to humans		no
E		4,08	Creates a fire hazard in natural ecosystems		yes
E		4,09	Is a shade tolerant plant at some stage of its life cycle		
E		4,10	Grows on infertile soils		yes
E		4,11	Climbing or smothering growth habit		no
E		4,12	Forms dense thickets		no
E	**5** *Plant*	5,01	Aquatic		no
C	*type*	5,02	Grass		no
E		5,03	Nitrogen fixing woody plant		no
C		5,04	Geophyte		no
C	**6** *Reproduction*	6,01	Evidence of substantial reproductive failure in native habitat		yes
C		6,02	Produces viable seed		yes
C		6,03	Hybridises naturally		
C		6,04	Self-fertilisation		yes
C		6,05	Requires specialist pollinators		no
C		6,06	Reproduction by vegetative propagation		no
C		6,07	Minimum generative time (years)		2
A	**7** *Dispersal*	7,01	Propagules likely to be dispersed unintentionally		no
C	*mechanisms*	7,02	Propagules dispersed intentionally by people		yes
A		7,03	Propagules likely to disperse as a produce contaminant		no
C		7,04	Propagules adapted to wind dispersal		yes
E		7,05	Propagules buoyant		
E		7,06	Propagules bird dispersed		no
C		7,07	Propagules dispersed by other animals (externally)		no
C		7,08	Propagules dispersed by other animals (internally)		no
C	**8** *Persistence*	8,01	Prolific seed production		
A	*attributes*	8,02	Evidence that a persistent propagule bank is formed (>1 yr)		
A		8,03	Well controlled by herbicides		
A		8,04	Tolerates or benefits from mutilation, cultivation or fire		yes
E		8,05	Effective natural enemies present in Australia		yes
	**Statistical summary of scoring**		Score partition:	Biogeography	-1
				Undesirable attributes	2
				Biology/ecology	-1
			Questions answered:	Biogeography	7
				Undesirable attributes	10
				Biology/ecology	19

A = agricultural, E = environmental, C = combined

Weed Risk Assessment report

20-Oct-17

**Botanical name:** *Banksia ericifolia*	**Outcome:** 0
**Common name:**	**Score:**
**Name of Assessor:**	**Agricultural** 0
	**Environmental** 0
	**Total** 11

	**A.**	**History/Biogeography**			
A	**1**	*Domestication/ cultivation*	1,01	Is the species highly domesticated? If answer is 'no' go to 2.01	no
C			1,02	Is species naturalised where grown?	yes
C			1,03	Does the species have weedy races?	
	**2**	*Climate and Distribution*	2,01	Species suited to Australian climates (0-low; 1-intermediate; 2-high)	2
			2,02	Quality of climate match data (0-low; 1-intermediate; 2-high)	2
C			2,03	Broad climate suitability (environmental versatility)	yes
C			2,04	Native or naturalised in regions with extended dry periods	
			2,05	Does the species have a history of repeated introductions outside its natural range?	yes
C	**3**	*Weed Elsewhere*	3,01	Naturalised beyond native range	yes
E			3,02	Garden/amenity/disturbance weed	no
A			3,03	Weed of agriculture/horticulture/forestry	no
E			3,04	Environmental weed	
			3,05	Congeneric weed	yes
	**B.**	**Biology/Ecology**			
A	**4**	*Undesirable traits*	4,01	Produces spines, thorns or burrs	no
C			4,02	Allelopathic	no
C			4,03	Parasitic	no
A			4,04	Unpalatable to grazing animals	no
C			4,05	Toxic to animals	no
C			4,06	Host for recognised pests and pathogens	yes
C			4,07	Causes allergies or is otherwise toxic to humans	no
E			4,08	Creates a fire hazard in natural ecosystems	yes
E			4,09	Is a shade tolerant plant at some stage of its life cycle	
E			4,10	Grows on infertile soils	yes
E			4,11	Climbing or smothering growth habit	no
E			4,12	Forms dense thickets	yes
E	**5**	*Plant type*	5,01	Aquatic	no
C			5,02	Grass	no
E			5,03	Nitrogen fixing woody plant	no
C			5,04	Geophyte	no
C	**6**	*Reproduction*	6,01	Evidence of substantial reproductive failure in native habitat	yes
C			6,02	Produces viable seed	yes
C			6,03	Hybridises naturally	
C			6,04	Self-fertilisation	yes
C			6,05	Requires specialist pollinators	no
C			6,06	Reproduction by vegetative propagation	no
C			6,07	Minimum generative time (years)	3
A	**7**	*Dispersal mechanisms*	7,01	Propagules likely to be dispersed unintentionally	no
C			7,02	Propagules dispersed intentionally by people	yes
A			7,03	Propagules likely to disperse as a produce contaminant	no
C			7,04	Propagules adapted to wind dispersal	yes
E			7,05	Propagules buoyant	
E			7,06	Propagules bird dispersed	no
C			7,07	Propagules dispersed by other animals (externally)	
C			7,08	Propagules dispersed by other animals (internally)	
C	**8**	*Persistence attributes*	8,01	Prolific seed production	yes
A			8,02	Evidence that a persistent propagule bank is formed (>1 yr)	yes
A			8,03	Well controlled by herbicides	
A			8,04	Tolerates or benefits from mutilation, cultivation or fire	yes
E			8,05	Effective natural enemies present in Australia	yes

**Statistical summary of scoring**	Score partition:	Biogeography	5
		Undesirable attributes	3
		Biology/ecology	3
	Questions answered:	Biogeography	6
		Undesirable attributes	11
		Biology/ecology	19

A = agricultural, E = environmental, C = combined

Weed Risk Assessment report

20-Oct-17

	**Botanical name:** *Banksia formosa*			**Outcome:**	0
	**Common name:**			**Score:**	
	**Name of Assessor:**			**Agricultural**	0
				**Environmental**	0
	**A. History/Biogeography**			**Total**	3

A	**1**	*Domestication/ cultivation*	1,01	Is the species highly domesticated? If answer is 'no' go to 2.01	no
C			1,02	Is species naturalised where grown?	no
C			1,03	Does the species have weedy races?	
	**2**	*Climate and Distribution*	2,01	Species suited to Australian climates (0-low; 1-intermediate; 2-high)	2
			2,02	Quality of climate match data (0-low; 1-intermediate; 2-high)	2
C			2,03	Broad climate suitability (environmental versatility)	no
C			2,04	Native or naturalised in regions with extended dry periods	
			2,05	Does the species have a history of repeated introductions outside its natural range?	no
C	**3**	*Weed Elsewhere*	3,01	Naturalised beyond native range	yes
E			3,02	Garden/amenity/disturbance weed	no
A			3,03	Weed of agriculture/horticulture/forestry	no
E			3,04	Environmental weed	
			3,05	Congeneric weed	yes
	**B. Biology/Ecology**				
A	**4**	*Undesirable traits*	4,01	Produces spines, thorns or burrs	no
C			4,02	Allelopathic	no
C			4,03	Parasitic	no
A			4,04	Unpalatable to grazing animals	no
C			4,05	Toxic to animals	
C			4,06	Host for recognised pests and pathogens	no
C			4,07	Causes allergies or is otherwise toxic to humans	
E			4,08	Creates a fire hazard in natural ecosystems	yes
E			4,09	Is a shade tolerant plant at some stage of its life cycle	
E			4,10	Grows on infertile soils	yes
E			4,11	Climbing or smothering growth habit	no
E			4,12	Forms dense thickets	no
E	**5**	*Plant type*	5,01	Aquatic	no
C			5,02	Grass	no
E			5,03	Nitrogen fixing woody plant	no
C			5,04	Geophyte	no
C	**6**	*Reproduction*	6,01	Evidence of substantial reproductive failure in native habitat	
C			6,02	Produces viable seed	yes
C			6,03	Hybridises naturally	
C			6,04	Self-fertilisation	yes
C			6,05	Requires specialist pollinators	
C			6,06	Reproduction by vegetative propagation	no
C			6,07	Minimum generative time (years)	
A	**7**	*Dispersal mechanisms*	7,01	Propagules likely to be dispersed unintentionally	no
C			7,02	Propagules dispersed intentionally by people	yes
A			7,03	Propagules likely to disperse as a produce contaminant	no
C			7,04	Propagules adapted to wind dispersal	no
E			7,05	Propagules buoyant	
E			7,06	Propagules bird dispersed	no
C			7,07	Propagules dispersed by other animals (externally)	
C			7,08	Propagules dispersed by other animals (internally)	
C	**8**	*Persistence attributes*	8,01	Prolific seed production	
A			8,02	Evidence that a persistent propagule bank is formed (>1 yr)	yes
A			8,03	Well controlled by herbicides	
A			8,04	Tolerates or benefits from mutilation, cultivation or fire	
E			8,05	Effective natural enemies present in Australia	yes

**Statistical summary of scoring**	Score partition:	Biogeography	4
		Undesirable attributes	1
		Biology/ecology	-2
	Questions answered:	Biogeography	6
		Undesirable attributes	9
		Biology/ecology	14

A = agricultural, E = environmental, C = combined

Weed Risk Assessment report

20-Oct-17

	**Botanical name:** *Banksia hookeriana*		**Outcome:**	0
	**Common name:**		**Score:**	
	**Name of Assessor:**		**Agricultural**	0
			**Environmental**	0
			**Total**	4

	**A.**	**History/Biogeography**			
A	**1**	*Domestication/*	1,01	Is the species highly domesticated? If answer is 'no' go to 2.01	no
C		*cultivation*	1,02	Is species naturalised where grown?	no
C			1,03	Does the species have weedy races?	
	**2**	*Climate and*	2,01	Species suited to Australian climates (0-low; 1-intermediate; 2-high)	2
		Distribution	2,02	Quality of climate match data (0-low; 1-intermediate; 2-high)	2
C			2,03	Broad climate suitability (environmental versatility)	no
C			2,04	Native or naturalised in regions with extended dry periods	
			2,05	Does the species have a history of repeated introductions outside its natural range?	no
C	**3**	*Weed*	3,01	Naturalised beyond native range	no
E		*Elsewhere*	3,02	Garden/amenity/disturbance weed	no
A			3,03	Weed of agriculture/horticulture/forestry	no
E			3,04	Environmental weed	no
			3,05	Congeneric weed	no
	**B.**	**Biology/Ecology**			
A	**4**	*Undesirable*	4,01	Produces spines, thorns or burrs	no
C		*traits*	4,02	Allelopathic	no
C			4,03	Parasitic	no
A			4,04	Unpalatable to grazing animals	no
C			4,05	Toxic to animals	
C			4,06	Host for recognised pests and pathogens	no
C			4,07	Causes allergies or is otherwise toxic to humans	
E			4,08	Creates a fire hazard in natural ecosystems	yes
E			4,09	Is a shade tolerant plant at some stage of its life cycle	
E			4,10	Grows on infertile soils	yes
E			4,11	Climbing or smothering growth habit	no
E			4,12	Forms dense thickets	no
E	**5**	*Plant*	5,01	Aquatic	no
C		*type*	5,02	Grass	no
E			5,03	Nitrogen fixing woody plant	no
C			5,04	Geophyte	no
C	**6**	*Reproduction*	6,01	Evidence of substantial reproductive failure in native habitat	
C			6,02	Produces viable seed	yes
C			6,03	Hybridises naturally	
C			6,04	Self-fertilisation	yes
C			6,05	Requires specialist pollinators	
C			6,06	Reproduction by vegetative propagation	no
C			6,07	Minimum generative time (years)	
A	**7**	*Dispersal*	7,01	Propagules likely to be dispersed unintentionally	no
C		*mechanisms*	7,02	Propagules dispersed intentionally by people	yes
A			7,03	Propagules likely to disperse as a produce contaminant	no
C			7,04	Propagules adapted to wind dispersal	yes
E			7,05	Propagules buoyant	
E			7,06	Propagules bird dispersed	
C			7,07	Propagules dispersed by other animals (externally)	
C			7,08	Propagules dispersed by other animals (internally)	
C	**8**	*Persistence*	8,01	Prolific seed production	yes
A		*attributes*	8,02	Evidence that a persistent propagule bank is formed (>1 yr)	yes
A			8,03	Well controlled by herbicides	
A			8,04	Tolerates or benefits from mutilation, cultivation or fire	yes
E			8,05	Effective natural enemies present in Australia	yes

**Statistical summary of scoring**	Score partition:	Biogeography	0
		Undesirable attributes	1
		Biology/ecology	3
	Questions answered:	Biogeography	7
		Undesirable attributes	9
		Biology/ecology	15

A = agricultural, E = environmental, C = combined

Weed Risk Assessment report

20-Oct-17

	**Botanical name:** *Banksia integrifolia*			**Outcome:**	0
	**Common name:**			**Score:**	
	**Name of Assessor:**			**Agricultural**	0
				**Environmental**	0
				**Total**	13
	**A. History/Biogeography**				
A	**1**	*Domestication/ cultivation*	1,01	Is the species highly domesticated? If answer is 'no' go to 2.01	yes
C			1,02	Is species naturalised where grown?	yes
C			1,03	Does the species have weedy races?	yes
	**2**	*Climate and Distribution*	2,01	Species suited to Australian climates (0-low; 1-intermediate; 2-high)	2
			2,02	Quality of climate match data (0-low; 1-intermediate; 2-high)	2
C			2,03	Broad climate suitability (environmental versatility)	yes
C			2,04	Native or naturalised in regions with extended dry periods	
			2,05	Does the species have a history of repeated introductions outside its natural range?	yes
C	**3**	*Weed Elsewhere*	3,01	Naturalised beyond native range	yes
E			3,02	Garden/amenity/disturbance weed	no
A			3,03	Weed of agriculture/horticulture/forestry	no
E			3,04	Environmental weed	
			3,05	Congeneric weed	yes
	**B. Biology/Ecology**				
A	**4**	*Undesirable traits*	4,01	Produces spines, thorns or burrs	no
C			4,02	Allelopathic	no
C			4,03	Parasitic	no
A			4,04	Unpalatable to grazing animals	
C			4,05	Toxic to animals	
C			4,06	Host for recognised pests and pathogens	no
C			4,07	Causes allergies or is otherwise toxic to humans	
E			4,08	Creates a fire hazard in natural ecosystems	yes
E			4,09	Is a shade tolerant plant at some stage of its life cycle	
E			4,10	Grows on infertile soils	yes
E			4,11	Climbing or smothering growth habit	no
E			4,12	Forms dense thickets	yes
E	**5**	*Plant type*	5,01	Aquatic	no
C			5,02	Grass	no
E			5,03	Nitrogen fixing woody plant	yes
C			5,04	Geophyte	no
C	**6**	*Reproduction*	6,01	Evidence of substantial reproductive failure in native habitat	
C			6,02	Produces viable seed	yes
C			6,03	Hybridises naturally	yes
C			6,04	Self-fertilisation	yes
C			6,05	Requires specialist pollinators	no
C			6,06	Reproduction by vegetative propagation	yes
C			6,07	Minimum generative time (years)	3
A	**7**	*Dispersal mechanisms*	7,01	Propagules likely to be dispersed unintentionally	no
C			7,02	Propagules dispersed intentionally by people	yes
A			7,03	Propagules likely to disperse as a produce contaminant	no
C			7,04	Propagules adapted to wind dispersal	yes
E			7,05	Propagules buoyant	
E			7,06	Propagules bird dispersed	
C			7,07	Propagules dispersed by other animals (externally)	
C			7,08	Propagules dispersed by other animals (internally)	
C	**8**	*Persistence attributes*	8,01	Prolific seed production	yes
A			8,02	Evidence that a persistent propagule bank is formed (>1 yr)	yes
A			8,03	Well controlled by herbicides	yes
A			8,04	Tolerates or benefits from mutilation, cultivation or fire	yes
E			8,05	Effective natural enemies present in Australia	no
	**Statistical summary of scoring**		Score partition:	Biogeography	2
				Undesirable attributes	3
				Biology/ecology	8
			Questions answered:	Biogeography	6
				Undesirable attributes	8
				Biology/ecology	19

A = agricultural, E = environmental, C = combined

Weed Risk Assessment report

20-Oct-17

	**Botanical name:** *Banksia menziesii*		**Outcome:**	0
	**Common name:**		**Score:**	
	**Name of Assessor:**		**Agricultural**	0
			**Environmental**	0
			**Total**	5

	**A.**	**History/Biogeography**			
A	**1**	*Domestication/ cultivation*	1,01	Is the species highly domesticated? If answer is 'no' go to 2.01	no
C			1,02	Is species naturalised where grown?	no
C			1,03	Does the species have weedy races?	
	**2**	*Climate and Distribution*	2,01	Species suited to Australian climates (0-low; 1-intermediate; 2-high)	2
			2,02	Quality of climate match data (0-low; 1-intermediate; 2-high)	2
C			2,03	Broad climate suitability (environmental versatility)	yes
C			2,04	Native or naturalised in regions with extended dry periods	
			2,05	Does the species have a history of repeated introductions outside its natural range?	yes
C	**3**	*Weed Elsewhere*	3,01	Naturalised beyond native range	no
E			3,02	Garden/amenity/disturbance weed	no
A			3,03	Weed of agriculture/horticulture/forestry	no
E			3,04	Environmental weed	no
			3,05	Congeneric weed	no
	**B.**	**Biology/Ecology**			
A	**4**	*Undesirable traits*	4,01	Produces spines, thorns or burrs	no
C			4,02	Allelopathic	no
C			4,03	Parasitic	no
A			4,04	Unpalatable to grazing animals	
C			4,05	Toxic to animals	
C			4,06	Host for recognised pests and pathogens	no
C			4,07	Causes allergies or is otherwise toxic to humans	
E			4,08	Creates a fire hazard in natural ecosystems	yes
E			4,09	Is a shade tolerant plant at some stage of its life cycle	
E			4,10	Grows on infertile soils	yes
E			4,11	Climbing or smothering growth habit	no
E			4,12	Forms dense thickets	no
E	**5**	*Plant type*	5,01	Aquatic	no
C			5,02	Grass	no
E			5,03	Nitrogen fixing woody plant	no
C			5,04	Geophyte	no
C	**6**	*Reproduction*	6,01	Evidence of substantial reproductive failure in native habitat	yes
C			6,02	Produces viable seed	yes
C			6,03	Hybridises naturally	
C			6,04	Self-fertilisation	yes
C			6,05	Requires specialist pollinators	no
C			6,06	Reproduction by vegetative propagation	no
C			6,07	Minimum generative time (years)	
A	**7**	*Dispersal mechanisms*	7,01	Propagules likely to be dispersed unintentionally	no
C			7,02	Propagules dispersed intentionally by people	yes
A			7,03	Propagules likely to disperse as a produce contaminant	no
C			7,04	Propagules adapted to wind dispersal	yes
E			7,05	Propagules buoyant	
E			7,06	Propagules bird dispersed	
C			7,07	Propagules dispersed by other animals (externally)	
C			7,08	Propagules dispersed by other animals (internally)	
C	**8**	*Persistence attributes*	8,01	Prolific seed production	yes
A			8,02	Evidence that a persistent propagule bank is formed (>1 yr)	yes
A			8,03	Well controlled by herbicides	
A			8,04	Tolerates or benefits from mutilation, cultivation or fire	yes
E			8,05	Effective natural enemies present in Australia	yes

**Statistical summary of scoring**	Score partition:	Biogeography	-1
		Undesirable attributes	2
		Biology/ecology	4
	Questions answered:	Biogeography	7
		Undesirable attributes	8
		Biology/ecology	17

A = agricultural, E = environmental, C = combined

Weed Risk Assessment report

20-Oct-17

**Botanical name:** *Banksia prionotes*			**Outcome:**	0
**Common name:**			**Score:**	
**Name of Assessor:**			**Agricultural**	0
			**Environmental**	0
			**Total**	1

	**A.**	**History/Biogeography**			
A	**1**	*Domestication/*	1,01	Is the species highly domesticated? If answer is 'no' go to 2.01	no
C		*cultivation*	1,02	Is species naturalised where grown?	no
C			1,03	Does the species have weedy races?	
	**2**	*Climate and*	2,01	Species suited to Australian climates (0-low; 1-intermediate; 2-high)	2
		Distribution	2,02	Quality of climate match data (0-low; 1-intermediate; 2-high)	2
C			2,03	Broad climate suitability (environmental versatility)	yes
C			2,04	Native or naturalised in regions with extended dry periods	
			2,05	Does the species have a history of repeated introductions outside its natural range?	yes
C	**3**	*Weed*	3,01	Naturalised beyond native range	no
E		*Elsewhere*	3,02	Garden/amenity/disturbance weed	no
A			3,03	Weed of agriculture/horticulture/forestry	no
E			3,04	Environmental weed	no
			3,05	Congeneric weed	no
	**B.**	**Biology/Ecology**			
A	**4**	*Undesirable*	4,01	Produces spines, thorns or burrs	no
C		*traits*	4,02	Allelopathic	no
C			4,03	Parasitic	no
A			4,04	Unpalatable to grazing animals	no
C			4,05	Toxic to animals	
C			4,06	Host for recognised pests and pathogens	no
C			4,07	Causes allergies or is otherwise toxic to humans	no
E			4,08	Creates a fire hazard in natural ecosystems	yes
E			4,09	Is a shade tolerant plant at some stage of its life cycle	
E			4,10	Grows on infertile soils	yes
E			4,11	Climbing or smothering growth habit	no
E			4,12	Forms dense thickets	no
E	**5**	*Plant*	5,01	Aquatic	no
C		*type*	5,02	Grass	no
E			5,03	Nitrogen fixing woody plant	no
C			5,04	Geophyte	no
C	**6**	*Reproduction*	6,01	Evidence of substantial reproductive failure in native habitat	yes
C			6,02	Produces viable seed	yes
C			6,03	Hybridises naturally	
C			6,04	Self-fertilisation	yes
C			6,05	Requires specialist pollinators	no
C			6,06	Reproduction by vegetative propagation	no
C			6,07	Minimum generative time (years)	
A	**7**	*Dispersal*	7,01	Propagules likely to be dispersed unintentionally	no
C		*mechanisms*	7,02	Propagules dispersed intentionally by people	yes
A			7,03	Propagules likely to disperse as a produce contaminant	no
C			7,04	Propagules adapted to wind dispersal	yes
E			7,05	Propagules buoyant	
E			7,06	Propagules bird dispersed	no
C			7,07	Propagules dispersed by other animals (externally)	no
C			7,08	Propagules dispersed by other animals (internally)	no
C	**8**	*Persistence*	8,01	Prolific seed production	yes
A		*attributes*	8,02	Evidence that a persistent propagule bank is formed (>1 yr)	yes
A			8,03	Well controlled by herbicides	
A			8,04	Tolerates or benefits from mutilation, cultivation or fire	yes
E			8,05	Effective natural enemies present in Australia	yes

**Statistical summary of scoring**	Score partition:	Biogeography	-1
		Undesirable attributes	1
		Biology/ecology	1
	Questions answered:	Biogeography	7
		Undesirable attributes	10
		Biology/ecology	20

A = agricultural, E = environmental, C = combined

Weed Risk Assessment report

20-Oct-17

Botanical name: *Banksia quercifolia*
Common name:
Name of Assessor:

Outcome: 0
Score:
Agricultural 0
Environmental 0
Total 7

	**A.**	**History/Biogeography**			
A	**1**	*Domestication/*	1,01	Is the species highly domesticated? If answer is 'no' go to 2.01	no
C		*cultivation*	1,02	Is species naturalised where grown?	no
C			1,03	Does the species have weedy races?	
	**2**	*Climate and*	2,01	Species suited to Australian climates (0-low; 1-intermediate; 2-high)	2
		Distribution	2,02	Quality of climate match data (0-low; 1-intermediate; 2-high)	2
C			2,03	Broad climate suitability (environmental versatility)	yes
C			2,04	Native or naturalised in regions with extended dry periods	
			2,05	Does the species have a history of repeated introductions outside its natural range?	no
C	**3**	*Weed*	3,01	Naturalised beyond native range	no
E		*Elsewhere*	3,02	Garden/amenity/disturbance weed	no
A			3,03	Weed of agriculture/horticulture/forestry	no
E			3,04	Environmental weed	no
			3,05	Congeneric weed	no
	**B.**	**Biology/Ecology**			
A	**4**	*Undesirable*	4,01	Produces spines, thorns or burrs	no
C		*traits*	4,02	Allelopathic	no
C			4,03	Parasitic	no
A			4,04	Unpalatable to grazing animals	
C			4,05	Toxic to animals	
C			4,06	Host for recognised pests and pathogens	no
C			4,07	Causes allergies or is otherwise toxic to humans	
E			4,08	Creates a fire hazard in natural ecosystems	yes
E			4,09	Is a shade tolerant plant at some stage of its life cycle	
E			4,10	Grows on infertile soils	yes
E			4,11	Climbing or smothering growth habit	no
E			4,12	Forms dense thickets	yes
E	**5**	*Plant*	5,01	Aquatic	no
C		*type*	5,02	Grass	no
E			5,03	Nitrogen fixing woody plant	no
C			5,04	Geophyte	no
C	**6**	*Reproduction*	6,01	Evidence of substantial reproductive failure in native habitat	
C			6,02	Produces viable seed	yes
C			6,03	Hybridises naturally	
C			6,04	Self-fertilisation	
C			6,05	Requires specialist pollinators	no
C			6,06	Reproduction by vegetative propagation	yes
C			6,07	Minimum generative time (years)	3
A	**7**	*Dispersal*	7,01	Propagules likely to be dispersed unintentionally	no
C		*mechanisms*	7,02	Propagules dispersed intentionally by people	yes
A			7,03	Propagules likely to disperse as a produce contaminant	no
C			7,04	Propagules adapted to wind dispersal	yes
E			7,05	Propagules buoyant	
E			7,06	Propagules bird dispersed	
C			7,07	Propagules dispersed by other animals (externally)	
C			7,08	Propagules dispersed by other animals (internally)	
C	**8**	*Persistence*	8,01	Prolific seed production	
A		*attributes*	8,02	Evidence that a persistent propagule bank is formed (>1 yr)	yes
A			8,03	Well controlled by herbicides	
A			8,04	Tolerates or benefits from mutilation, cultivation or fire	yes
E			8,05	Effective natural enemies present in Australia	yes
	**Statistical summary of scoring**			Score partition: Biogeography	1
				Undesirable attributes	3
				Biology/ecology	3
				Questions answered: Biogeography	7
				Undesirable attributes	8
				Biology/ecology	15

A = agricultural, E = environmental, C = combined

Weed Risk Assessment report

20-Oct-17

	**Botanical name:** *Banksia serrata*			**Outcome:**	0
	**Common name:**			**Score:**	
	**Name of Assessor:**			**Agricultural**	0
				**Environmental**	0
	**A. History/Biogeography**			**Total**	2
A	**1** *Domestication/*	1,01	Is the species highly domesticated? If answer is 'no' go to 2.01		no
C	*cultivation*	1,02	Is species naturalised where grown?		no
C		1,03	Does the species have weedy races?		
	**2** *Climate and*	2,01	Species suited to Australian climates (0-low; 1-intermediate; 2-high)		2
	Distribution	2,02	Quality of climate match data (0-low; 1-intermediate; 2-high)		2
C		2,03	Broad climate suitability (environmental versatility)		no
C		2,04	Native or naturalised in regions with extended dry periods		
		2,05	Does the species have a history of repeated introductions outside its natural range?		no
C	**3** *Weed*	3,01	Naturalised beyond native range		
E	*Elsewhere*	3,02	Garden/amenity/disturbance weed		no
A		3,03	Weed of agriculture/horticulture/forestry		no
E		3,04	Environmental weed		
		3,05	Congeneric weed		
	**B. Biology/Ecology**				
A	**4** *Undesirable*	4,01	Produces spines, thorns or burrs		no
C	*traits*	4,02	Allelopathic		no
C		4,03	Parasitic		no
A		4,04	Unpalatable to grazing animals		
C		4,05	Toxic to animals		
C		4,06	Host for recognised pests and pathogens		no
C		4,07	Causes allergies or is otherwise toxic to humans		
E		4,08	Creates a fire hazard in natural ecosystems		yes
E		4,09	Is a shade tolerant plant at some stage of its life cycle		
E		4,10	Grows on infertile soils		yes
E		4,11	Climbing or smothering growth habit		no
E		4,12	Forms dense thickets		no
E	**5** *Plant*	5,01	Aquatic		no
C	*type*	5,02	Grass		no
E		5,03	Nitrogen fixing woody plant		no
C		5,04	Geophyte		no
C	**6** *Reproduction*	6,01	Evidence of substantial reproductive failure in native habitat		
C		6,02	Produces viable seed		yes
C		6,03	Hybridises naturally		
C		6,04	Self-fertilisation		yes
C		6,05	Requires specialist pollinators		
C		6,06	Reproduction by vegetative propagation		no
C		6,07	Minimum generative time (years)		
A	**7** *Dispersal*	7,01	Propagules likely to be dispersed unintentionally		no
C	*mechanisms*	7,02	Propagules dispersed intentionally by people		yes
A		7,03	Propagules likely to disperse as a produce contaminant		no
C		7,04	Propagules adapted to wind dispersal		yes
E		7,05	Propagules buoyant		
E		7,06	Propagules bird dispersed		no
C		7,07	Propagules dispersed by other animals (externally)		
C		7,08	Propagules dispersed by other animals (internally)		
C	**8** *Persistence*	8,01	Prolific seed production		
A	*attributes*	8,02	Evidence that a persistent propagule bank is formed (>1 yr)		
A		8,03	Well controlled by herbicides		
A		8,04	Tolerates or benefits from mutilation, cultivation or fire		
E		8,05	Effective natural enemies present in Australia		
	**Statistical summary**			Biogeography	0
	**of scoring**		Score partition:	Undesirable attributes	2
				Biology/ecology	0
				Biogeography	4
			Questions answered:	Undesirable attributes	8
				Biology/ecology	12

A = agricultural, E = environmental, C = combined

Weed Risk Assessment report

20-Oct-17

**Botanical name:** *Banksia speciosa*			**Outcome:**	0
**Common name:**			**Score:**	
**Name of Assessor:**			**Agricultural**	0
			**Environmental**	0
			**Total**	8

	**A.**	**History/Biogeography**			
A	**1**	*Domestication/ cultivation*	1,01	Is the species highly domesticated? If answer is 'no' go to 2.01	no
C			1,02	Is species naturalised where grown?	yes
C			1,03	Does the species have weedy races?	
	**2**	*Climate and Distribution*	2,01	Species suited to Australian climates (0-low; 1-intermediate; 2-high)	2
			2,02	Quality of climate match data (0-low; 1-intermediate; 2-high)	2
C			2,03	Broad climate suitability (environmental versatility)	
C			2,04	Native or naturalised in regions with extended dry periods	
			2,05	Does the species have a history of repeated introductions outside its natural range?	yes
C	**3**	*Weed Elsewhere*	3,01	Naturalised beyond native range	yes
E			3,02	Garden/amenity/disturbance weed	no
A			3,03	Weed of agriculture/horticulture/forestry	no
E			3,04	Environmental weed	no
			3,05	Congeneric weed	yes
	**B.**	**Biology/Ecology**			
A	**4**	*Undesirable traits*	4,01	Produces spines, thorns or burrs	no
C			4,02	Allelopathic	no
C			4,03	Parasitic	no
A			4,04	Unpalatable to grazing animals	
C			4,05	Toxic to animals	
C			4,06	Host for recognised pests and pathogens	yes
C			4,07	Causes allergies or is otherwise toxic to humans	no
E			4,08	Creates a fire hazard in natural ecosystems	yes
E			4,09	Is a shade tolerant plant at some stage of its life cycle	
E			4,10	Grows on infertile soils	yes
E			4,11	Climbing or smothering growth habit	no
E			4,12	Forms dense thickets	yes
E	**5**	*Plant type*	5,01	Aquatic	no
C			5,02	Grass	no
E			5,03	Nitrogen fixing woody plant	no
C			5,04	Geophyte	no
C	**6**	*Reproduction*	6,01	Evidence of substantial reproductive failure in native habitat	yes
C			6,02	Produces viable seed	yes
C			6,03	Hybridises naturally	no
C			6,04	Self-fertilisation	yes
C			6,05	Requires specialist pollinators	no
C			6,06	Reproduction by vegetative propagation	
C			6,07	Minimum generative time (years)	
A	**7**	*Dispersal mechanisms*	7,01	Propagules likely to be dispersed unintentionally	no
C			7,02	Propagules dispersed intentionally by people	yes
A			7,03	Propagules likely to disperse as a produce contaminant	no
C			7,04	Propagules adapted to wind dispersal	yes
E			7,05	Propagules buoyant	
E			7,06	Propagules bird dispersed	no
C			7,07	Propagules dispersed by other animals (externally)	no
C			7,08	Propagules dispersed by other animals (internally)	no
C	**8**	*Persistence attributes*	8,01	Prolific seed production	yes
A			8,02	Evidence that a persistent propagule bank is formed (>1 yr)	yes
A			8,03	Well controlled by herbicides	yes
A			8,04	Tolerates or benefits from mutilation, cultivation or fire	yes
E			8,05	Effective natural enemies present in Australia	yes

**Statistical summary of scoring**	Score partition:	Biogeography	4
		Undesirable attributes	4
		Biology/ecology	0
	Questions answered:	Biogeography	6
		Undesirable attributes	9
		Biology/ecology	21

A = agricultural, E = environmental, C = combined

Weed Risk Assessment report

20-Oct-17

	**Botanical name:** *Banksia sphaerocarpa*			**Outcome:**	0
	**Common name:**			**Score:**	
	**Name of Assessor:**			**Agricultural**	0
				**Environmental**	0
				**Total**	3
	**A. History/Biogeography**				
A	**1** *Domestication/ cultivation*	1,01	Is the species highly domesticated? If answer is 'no' go to 2.01		no
C		1,02	Is species naturalised where grown?		no
C		1,03	Does the species have weedy races?		
	**2** *Climate and Distribution*	2,01	Species suited to Australian climates (0-low; 1-intermediate; 2-high)		2
		2,02	Quality of climate match data (0-low; 1-intermediate; 2-high)		2
C		2,03	Broad climate suitability (environmental versatility)		no
C		2,04	Native or naturalised in regions with extended dry periods		
		2,05	Does the species have a history of repeated introductions outside its natural range?		no
C	**3** *Weed Elsewhere*	3,01	Naturalised beyond native range		
E		3,02	Garden/amenity/disturbance weed		no
A		3,03	Weed of agriculture/horticulture/forestry		no
E		3,04	Environmental weed		
		3,05	Congeneric weed		
	**B. Biology/Ecology**				
A	**4** *Undesirable traits*	4,01	Produces spines, thorns or burrs		no
C		4,02	Allelopathic		no
C		4,03	Parasitic		no
A		4,04	Unpalatable to grazing animals		
C		4,05	Toxic to animals		
C		4,06	Host for recognised pests and pathogens		no
C		4,07	Causes allergies or is otherwise toxic to humans		
E		4,08	Creates a fire hazard in natural ecosystems		yes
E		4,09	Is a shade tolerant plant at some stage of its life cycle		
E		4,10	Grows on infertile soils		yes
E		4,11	Climbing or smothering growth habit		no
E		4,12	Forms dense thickets		no
E	**5** *Plant type*	5,01	Aquatic		no
C		5,02	Grass		no
E		5,03	Nitrogen fixing woody plant		no
C		5,04	Geophyte		no
C	**6** *Reproduction*	6,01	Evidence of substantial reproductive failure in native habitat		
C		6,02	Produces viable seed		yes
C		6,03	Hybridises naturally		
C		6,04	Self-fertilisation		yes
C		6,05	Requires specialist pollinators		
C		6,06	Reproduction by vegetative propagation		no
C		6,07	Minimum generative time (years)		
A	**7** *Dispersal mechanisms*	7,01	Propagules likely to be dispersed unintentionally		
C		7,02	Propagules dispersed intentionally by people		yes
A		7,03	Propagules likely to disperse as a produce contaminant		no
C		7,04	Propagules adapted to wind dispersal		yes
E		7,05	Propagules buoyant		
E		7,06	Propagules bird dispersed		no
C		7,07	Propagules dispersed by other animals (externally)		
C		7,08	Propagules dispersed by other animals (internally)		
C	**8** *Persistence attributes*	8,01	Prolific seed production		
A		8,02	Evidence that a persistent propagule bank is formed (>1 yr)		
A		8,03	Well controlled by herbicides		
A		8,04	Tolerates or benefits from mutilation, cultivation or fire		
E		8,05	Effective natural enemies present in Australia		
	**Statistical summary of scoring**		Score partition:	Biogeography	0
				Undesirable attributes	2
				Biology/ecology	1
			Questions answered:	Biogeography	4
				Undesirable attributes	8
				Biology/ecology	11

A = agricultural, E = environmental, C = combined

Weed Risk Assessment report

20-Oct-17

	**Botanical name:** *Banksia spinulosa*			**Outcome:**	0
	**Common name:**			**Score:**	
	**Name of Assessor:**			**Agricultural**	0
	**A. History/Biogeography**			**Environmental**	0
				**Total**	9
A	**1** *Domestication/ cultivation*	1,01	Is the species highly domesticated? If answer is 'no' go to 2.01		no
C		1,02	Is species naturalised where grown?		no
C		1,03	Does the species have weedy races?		
	**2** *Climate and Distribution*	2,01	Species suited to Australian climates (0-low; 1-intermediate; 2-high)		2
		2,02	Quality of climate match data (0-low; 1-intermediate; 2-high)		2
C		2,03	Broad climate suitability (environmental versatility)		yes
C		2,04	Native or naturalised in regions with extended dry periods		
		2,05	Does the species have a history of repeated introductions outside its natural range?		no
C	**3** *Weed Elsewhere*	3,01	Naturalised beyond native range		no
E		3,02	Garden/amenity/disturbance weed		no
A		3,03	Weed of agriculture/horticulture/forestry		no
E		3,04	Environmental weed		
		3,05	Congeneric weed		yes
	**B. Biology/Ecology**				
A	**4** *Undesirable traits*	4,01	Produces spines, thorns or burrs		no
C		4,02	Allelopathic		no
C		4,03	Parasitic		no
A		4,04	Unpalatable to grazing animals		
C		4,05	Toxic to animals		
C		4,06	Host for recognised pests and pathogens		no
C		4,07	Causes allergies or is otherwise toxic to humans		
E		4,08	Creates a fire hazard in natural ecosystems		yes
E		4,09	Is a shade tolerant plant at some stage of its life cycle		
E		4,10	Grows on infertile soils		yes
E		4,11	Climbing or smothering growth habit		no
E		4,12	Forms dense thickets		no
E	**5** *Plant type*	5,01	Aquatic		no
C		5,02	Grass		no
E		5,03	Nitrogen fixing woody plant		no
C		5,04	Geophyte		no
C	**6** *Reproduction*	6,01	Evidence of substantial reproductive failure in native habitat		
C		6,02	Produces viable seed		yes
C		6,03	Hybridises naturally		
C		6,04	Self-fertilisation		yes
C		6,05	Requires specialist pollinators		no
C		6,06	Reproduction by vegetative propagation		
C		6,07	Minimum generative time (years)		2
A	**7** *Dispersal mechanisms*	7,01	Propagules likely to be dispersed unintentionally		no
C		7,02	Propagules dispersed intentionally by people		yes
A		7,03	Propagules likely to disperse as a produce contaminant		no
C		7,04	Propagules adapted to wind dispersal		yes
E		7,05	Propagules buoyant		
E		7,06	Propagules bird dispersed		
C		7,07	Propagules dispersed by other animals (externally)		
C		7,08	Propagules dispersed by other animals (internally)		
C	**8** *Persistence attributes*	8,01	Prolific seed production		
A		8,02	Evidence that a persistent propagule bank is formed (>1 yr)		yes
A		8,03	Well controlled by herbicides		
A		8,04	Tolerates or benefits from mutilation, cultivation or fire		yes
E		8,05	Effective natural enemies present in Australia		
	**Statistical summary of scoring**		Score partition:	Biogeography	3
				Undesirable attributes	2
				Biology/ecology	4
			Questions answered:	Biogeography	6
				Undesirable attributes	8
				Biology/ecology	14

A = agricultural, E = environmental, C = combined

Cowling, R. M., Lamont, B. B., & Pierce, S. M. (1987). SEED BANK DYNAMICS OF 4 COOCCURRING BANKSIA SPECIES. *Journal of Ecology*, *75*(2), 289–302. Journal Article. https://doi.org/10.2307/2260419

Fuss, A. M., Pattison, S. J., Aspinall, D., & Sedgley, M. (1992). Shoot growth in relation to cut flower production of Banksia coccinea and Banksia menziesii (Proteaceae). *Scientia Horticulturae*, *49*, 323–334.

Fuss, A. M., & Sedgley, M. (1991). Pollen tube growth and seed set of Banksia coccinea R.Br. (Proteaceae). *Ann. Bot.*, *68*, 377–384.

Geerts, S., Moodley, D., Gaertner, M., Le Roux, J. J., McGeoch, M. A., Muofhe, C., … Wilson, J. R. U. (2013). The absence of fire can cause a lag phase: The invasion dynamics of Banksia ericifolia Proteaceae. *Austral Ecology*, *38*, 931–941. article.

Hanley, M. E., & Lamont, B. B. (2001). Herbivory, serotiny and seedling defence in Western Australian Proteaceae. *Oecologia*, *126*(3), 409–417. article. https://doi.org/10.1007/s004420000538

He, T., Lamont, B. B., Krauss, S. L., Enright, N. J., & Miller, B. P. (2009). Long-distance dispersal of seeds in the fire-tolerant shrub Banksia attenuata. *Ecography*, *32*(4), 571–580. https://doi.org/10.1111/j.1600-0587.2008.05689.x

Lamont, B. B., & Groom, P. (998). Seed and Seedling Biology of the Woody-fruited Proteaceae. *Australian Journal of Botany1*, *46*, 387–406.

McCredie, T. A., Dixon, K. W., & Sivasithamparam, K. (1985). Variability in in the Resistance of Banksia L.F SSpecies to Phytophthora cinnamomi. *Australian Journal of Botany*, *33*(6), 629–637. article. https://doi.org/10.1071/bt9850629

Moodley, D., Geerts, S., Rebelo, T., Richardson, D. M., & Wilson, J. R. U. (2014). Site-specific conditions influence plant naturalization: The case of alien Proteaceae in South Africa. *Acta Oecologica*, *59*, 62–71. https://doi.org/10.1016/j.actao.2014.05.005

Moodley, D., Geerts, S., Richardson, D. M., & Wilson, J. R. U. (2013). Different traits determine introduction, and invasion success naturalization invasion success in woody plants: Proteaceae as a test case. *Plos One*, *8*(9), e75078. article. https://doi.org/10.1371/journal.pone.0075078

Moodley, D., Geerts, S., Richardson, D. M., & Wilson, J. R. U. (2016). The importance of pollinators and autonomous self-fertilization in the early stages of plant invasions: Banksia and Hakea (Proteaceae) as case studies. *Plant Biology*, *18*(1), 124–131. https://doi.org/10.1111/plb.12334

Proteas of Hawaii. (n.d.). Retrieved April 6, 2017, from https://www.proteasofhawaii.com/shopcontent.asp?type=flowerfacts

Richardson, D. M., Cowling, R. M., Lamont, B. B., & van Hensbergen, H. J. (1995). Coexistence of Banksia species in southwestern Australia: the role of regional and local processes. *Journal of Vegetation Science*, *6*(3), 329–342. https://doi.org/10.2307/3236232

Species, S. B. D. of F. C. B. (1987). Seed Bank Dynamics of Four Co-Occurring Banksia Species Author (s): R . M . Cowling , Byron B . Lamont and S . M . Pierce Published by : British Ecological Society Stable URL : http://www.jstor.org/stable/2260419 REFERENCES Linked references are availab. *Journal of Ecology*, *75*(2), 289–302.

Tynan, K. M., Scott, E. S., & Sedgley, M. (1998). Evaluation of Banksia species for response to

Phytophthora infection. *Plant Pathology*, *47*(4), 446–455. Retrieved from <Go to ISI>://000075458700009

WHELAN, R. J., & MAIN, A. R. (1979). Insect grazing and pos fire plant succession in south west Australian woodland. *Australian Journal of Ecology*, *4*(4), 387–398. https://doi.org/10.1111/j.1442-9993.1979.tb01566.x
