# Supplementary material for: Relationship of alien species continues in a foreign land: The case of Phytophthora and Australian Banksia (Proteaceae) in South African Fynbos
Source: Ecol Evol. 2022 Jul 14;12(7):10.1002/ece3.9100. doi: 10.1002/ece3.9100 (PMC9280440; doi:10.1002/ece3.9100)
Supplement: Supplementary file 2 — Table S1 [file ECE3-12--s002.docx]

Table S1: The table below shows summary data obtained from the soil nutrients analyses from *Banksia* localities in the Cape Floristic Region.

| **Species** | **pH (KCl)** | **Resist. (Ohm)** | **H+** | **Stone (Vol %)** | **P (mg/kg)** | **K** | **Ex. cations (cmol(+)/kg)** | | | | **Cu** | **Zn** | **Mn** | **B** | **Fe mg/kg** | **C** | **N** | **NO3-N** | **NH4-N** | **Clay** | **Silt** | **Sand** |
| --- | --- | --- | --- | --- | --- | --- | --- | --- | --- | --- | --- | --- | --- | --- | --- | --- | --- | --- | --- | --- | --- | --- |
|  |  |  | **(cmol/kg)** |  | **Bray II** | **mg/kg** | **Na** | **K** | **Ca** | **Mg** | **mg/kg** |  |  |  |  | **%** | **%** | **mg/kg** | **mg/kg** | **%** | **%** | **%** |
| ***B. baxteri*** | 4.1 | 2350 | 1.21 | 1 | 3 | 33 | 0.24 | 0.08 | 1.64 | 0.66 | 0.1 | 0.3 | 0.5 | 0.13 | 90 | 1.50 | 0.056 | 0.74 | 6.84 | 5 | 14 | 81 |
| ***B. speciosa*** | 5.1 | 3890 | 0.75 | 1 | 2 | 52 | 0.15 | 0.13 | 1.54 | 0.65 | 0.1 | 0.2 | 0.5 | 0.19 | 61 | 1.46 | 0.059 | 0.48 | 6.22 | 7 | 14 | 79 |
| ***B. spinulosa*** | 4.6 | 4080 | 0.70 | 2 | 1 | 38 | 0.12 | 0.10 | 0.76 | 0.41 | 0.1 | 0.2 | 0.4 | 0.06 | 42 | 0.94 | 0.054 | 0.64 | 5.03 | 7 | 12 | 81 |
| ***B. formosa*** | 4.5 | 4360 | 1.08 | 1 | 1 | 44 | 0.09 | 0.11 | 0.63 | 0.31 | 0.1 | 0.2 | 0.2 | 0.05 | 61 | 1.44 | 0.064 | 0.65 | 5.86 | 9 | 12 | 79 |
| ***B. serrata*** | 4.8 | 6010 | 0.67 | 1 | 7 | 25 | 0.07 | 0.07 | 0.90 | 0.32 | 0.1 | 0.2 | 0.7 | 0.10 | 88 | 0.84 | 0.058 | 0.46 | 5.70 | 5 | 6 | 89 |
| ***B. integrifolia*** | 5.2 | 1080 | 0.71 | 5 | 3 | 56 | 0.68 | 0.14 | 1.95 | 1.22 | 0.1 | 0.2 | 2.4 | 0.57 | 72 | 2.12 | 0.067 | 0.48 | 7.23 | 7 | 6 | 87 |
| ***B. coccinea*** | 4.8 | 1760 | 0.78 | 1 | 1 | 35 | 0.22 | 0.09 | 1.36 | 0.54 | 0.1 | 0.2 | 0.3 | 0.13 | 89 | 1.08 | 0.057 | 0.64 | 4.84 | 7 | 12 | 81 |
| ***B. hookeriana*** | 4.7 | 1670 | 0.83 | 1 | 2 | 41 | 0.16 | 0.11 | 1.64 | 0.63 | 0.1 | 0.2 | 0.4 | 0.05 | 56 | 1.37 | 0.062 | 0.81 | 6.22 | 7 | 18 | 75 |
| ***B. ericifolia*** | 4.8 | 1890 | 0.66 | 1 | 1 | 41 | 0.19 | 0.11 | 1.02 | 0.60 | 0.1 | 0.2 | 0.7 | 0.03 | 72 | 1.39 | 0.064 | 0.46 | 6.94 | 9 | 8 | 83 |
| ***B. prionotes*** | 4.0 | 7530 | 0.90 | 1 | 2 | 19 | 0.06 | 0.05 | 0.76 | 0.35 | 0.1 | 0.2 | 0.2 | 0.03 | 84 | 1.01 | 0.050 | 0.48 | 8.89 | 5 | 10 | 85 |
| ***B. manziesii*** | 4.6 | 7900 | 0.77 | 1 | 3 | 50 | 0.07 | 0.13 | 1.46 | 0.55 | 0.1 | 0.2 | 0.2 | 0.04 | 56 | 1.10 | 0.053 | 2.24 | 12.48 | 9 | 18 | 73 |
| ***B. integrifolia*** | 5.3 | 1190 | 0.58 | 6 | 3 | 31 | 0.4 | 0.08 | 2.31 | 1.17 | 0 | 0.2 | 0.4 | 0.87 | 57 | 1.75 | 0.067 | 2.57 | 7.08 | 5 | 6 | 89 |
| ***B. ericifolia*** | 4.5 | 710 | 0.7 | 50 | 3 | 28 | 0.42 | 0.07 | 1.44 | 0.86 | 0 | 0.2 | 0.2 | 0.22 | 179 | 1.59 | 0.057 | 1.46 | 4.85 | 9 | 4 | 87 |
| ***B. integrifolia*** | 3.8 | 1420 | 0.7 | 1 | 6 | 18 | 0.17 | 0.05 | 0.91 | 0.58 | 0 | 0.3 | 0.6 | 0.18 | 31 | 1.12 | 0.062 | 1.2 | 9.04 | 5 | 2 | 93 |
| **B. coccinea** | 4.9 | 5310 | 0.28 | 1 | 3 | 15 | 0.09 | 0.04 | 0.66 | 0.4 | 0 | 0.1 | 0.1 | 0.16 | 55 | 0.72 | 0.071 | 1.49 | 6.86 | 5 | 4 | 91 |
| ***B. baxteri*** | 4.5 | 2320 | 0.66 | 1 | 3 | 18 | 0.13 | 0.05 | 0.89 | 0.6 | 0 | 0.3 | 0.3 | 0.24 | 39 | 1.35 | 0.068 | 4.59 | 7.68 | 7 | 4 | 89 |
| ***B. hookeriana*** | 5 | 3840 | 0.34 | 1 | 3 | 26 | 0.1 | 0.07 | 1.62 | 0.51 | 0 | 0.3 | 0.1 | 0.17 | 29 | 1.02 | 0.07 | 1.77 | 5.52 | 5 | 4 | 91 |
| ***B. prionotes*** | 4.1 | 11570 | 0.24 | 1 | 1 | 14 | 0.06 | 0.04 | 0.39 | 0.34 | 0 | 0.2 | 0.1 | 0.1 | 17 | 0.62 | 0.063 | 1.39 | 5.37 | 5 | 4 | 91 |
| ***B. serrata*** | 4.7 | 5620 | 0.25 | 1 | 2 | 15 | 0.08 | 0.04 | 0.46 | 0.38 | 0 | 0.1 | 0 | 0.12 | 25 | 0.57 | 0.053 | 1.69 | 5.06 | 5 | 4 | 91 |
| ***B. speciosa*** | 4.5 | 2240 | 0.61 | 47 | 3 | 27 | 0.18 | 0.07 | 2.45 | 1.02 | 0.1 | 0.3 | 11.7 | 0.2 | 82 | 1.71 | 0.077 | 2.93 | 8.04 | 7 | 6 | 87 |
| ***B. formosa*** | 4.7 | 1270 | 0.64 | 58 | 3 | 45 | 0.28 | 0.11 | 2.6 | 1.3 | 0.1 | 0.2 | 5.8 | 0.32 | 138 | 1.56 | 0.089 | 2.05 | 9.61 | 9 | 6 | 85 |
| ***B. coccinea*** | 4.3 | 2920 | 0.83 | 70 | 1 | 24 | 0.15 | 0.06 | 2.67 | 0.92 | 0.1 | 0.3 | 6.6 | 0.17 | 91 | 1.43 | 0.082 | 1.89 | 7.27 | 7 | 6 | 87 |

Table S2. Risk analyses for all *Banksia* species in this study. Excel files, so online only.
